# Impacts of Combining *Steinernema carpocapsae* and *Bracon hebetor* Parasitism on *Galleria mellonella* Larvae

**DOI:** 10.3390/insects15080588

**Published:** 2024-08-01

**Authors:** Neama A. Aamer, Zeinab A. El-Moaty, Maria Augustyniak, Lamia M. El-Samad, Hanaa S. Hussein

**Affiliations:** 1 Department of Applied Entomology and Zoology, Faculty of Agriculture, Alexandria University, Alexandria 21545, Egypt; neama.aamer@alexu.edu.eg (N.A.A.);; 2 Biological Sciences Department, College of Science, King Faisal University, Al-Ahsaa 31982, Saudi Arabia; 3 Department of Zoology, Faculty of Science, Alexandria University, Moharam Bey, Alexandria 21511, Egypt; 4 Institute of Biology, Biotechnology and Environmental Protection, Faculty of Natural Sciences, University of Silesia in Katowice, Bankowa 9, 40-007 Katowice, Poland; maria.augustyniak@us.edu.pl

**Keywords:** greater wax moth, ectoparasite, nematoda, oxidative stress enzymes, DNA damage, annexin, scanning electron microscopy

## Abstract

**Simple Summary:**

The greater wax moth, *Galleria mellonella*, is a significant pest in apiculture. This investigation explores the individual and combined effects of the ectoparasite *Bracon hebetor* (B.h.) and the entomopathogenic nematode *Steinernema carpocapsae* (S.c.) on *G. mellonella* larvae. We found that control larvae exhibited higher glutathione peroxidase (GPx) and glutathione S-transferase (GST) activities compared to those treated with B.h., S.c., or the B.h. + S.c. combination. Conversely, malondialdehyde (MDA) levels displayed an opposite trend. Superoxide dismutase (SOD) activity was reduced in the B.h. and S.c. groups but significantly higher in the combined treatment. Cytochrome P450 activity increased in response to parasitism by *B. hebetor*. The Annexin V-FITC assay revealed decreased cell viability in parasitized groups (B.h. 79.4%, S.c. 77.3%, B.h. + S.c. 70.1%) compared to controls. DNA damage analysis demonstrated significant differences between groups, and SEM observations confirmed severe cuticle abnormalities or malformations in *G. mellonella* larvae.

**Abstract:**

The greater wax moth, *Galleria mellonella*, is a significant pest in apiculture and a well-established model organism for immunological and ecotoxicological studies. This investigation explores the individual and combined effects of the ectoparasite *Bracon hebetor* (B.h.) and the entomopathogenic nematode *Steinernema carpocapsae* (S.c.) on *G. mellonella* larvae. We evaluated the activity of oxidative stress enzymes, including superoxide dismutase (SOD), glutathione peroxidase (GPx), glutathione S-transferase (GST), malondialdehyde (MDA) levels, cytochrome P450 activity, cell viability using Annexin V-FITC, DNA damage via comet assay, and larval morphology through scanning electron microscopy (SEM). Control larvae exhibited higher GPx and GST activities compared to those treated with B.h., S.c., or the B.h. + S.c. combination. Conversely, MDA levels displayed the opposite trend. SOD activity was reduced in the B.h. and S.c. groups but significantly higher in the combined treatment. Cytochrome P450 activity increased in response to parasitism by *B. hebetor*. The Annexin V-FITC assay revealed decreased cell viability in parasitized groups (B.h. 79.4%, S.c. 77.3%, B.h. + S.c. 70.1%) compared to controls. DNA damage analysis demonstrated significant differences between groups, and SEM observations confirmed severe cuticle abnormalities or malformations in *G. mellonella* larvae. These findings highlight the complex interactions between *B. hebetor*, *S. carpocapsae*, and their host, *G. mellonella*. Additionally, they illuminate the intricate physiological responses triggered within the host larvae.

## 1. Introduction

The greater wax moth, *Galleria mellonella* (Linnaeus, 1758) (Pyralidae: Lepidoptera), is a globally recognized pest in apiculture, causing significant economic losses due to its larvae consuming honeycombs [1]. Because it is easy to handle, thrives in labs, and is inexpensive to raise, this organism is perfect for immune system and toxin studies [2,3]. This in vivo model offers several advantages, including low biohazard risk, ease of manipulation, affordability, and minimal space or infrastructure requirements, making it ethically acceptable and widely used in research [4]. Current approaches utilizing *G. mellonella* larvae primarily focus on host–pathogen interactions, with these larvae frequently employed as infection models to evaluate the efficacy of biological control agents before pre-clinical mammalian research [5].

Decades of widespread insecticide application have selected for resistant insect populations [6,7,8]. Various methods, including chemical treatments (e.g., aluminum phosphide, paradichlorobenzene, ethylene dibromide, carbon dioxide, and sulfur) and physical techniques (e.g., heat and cold treatments), have been employed to protect honeycombs from *G. mellonella* infestation [9]. However, biological control strategies utilizing natural enemies such as parasitic wasps are considered eco-friendly, sustainable, and harmless approaches for integrated pest management (IPM) [10,11]. These natural enemies can continuously breed as long as hosts are available, ensuring long-term pest population control [12].

Parasitic wasps, like *Bracon hebetor* (Say) (Hymenoptera: Braconidae), exhibit high dispersal rates and can locate hosts in hidden areas. They employ various strategies to regulate host physiology, including idiobiont and koinobiont lifestyles. Idiobionts permanently paralyze their hosts during parasitism, halting host growth and development, while koinobionts allow hosts to continue developing post-parasitism [13]. The host regulation process is complex, often resulting in significant physiological changes without causing immediate host death [14]. Venoms from at least six insect orders serve multiple purposes, including communication, protection, predation, and paralyzing hosts for progeny feeding [15,16,17,18,19,20]. In parasitoids, venom injections facilitate reproduction by preventing host interference during oviposition [21,22].

Within the Hymenoptera, parasitoids employ various host regulatory tactics, ranging from immune suppression to neuroendocrine disruptions affecting reproduction and development [23,24,25,26]. Parasitic wasps induce significant physiological changes in hosts through toxic secretions during egg laying, which can cause paralysis and rapid host death [21,26,27]. The idiobiont ectoparasitoid *B. hebetor* is particularly effective in the biocontrol of lepidopterous pests, triggering host defense mechanisms upon injury and penetration [28].

Entomopathogenic nematodes (EPNs), particularly those in the Steinernematidae and Heterorhabditidae families, also serve as biological control agents. These obligate parasites kill their insect hosts during part of their life cycle [29]. EPNs release symbiotic bacteria upon penetrating the host, with the bacteria proliferating and killing the host within 24–48 h [30]. EPNs are environmentally safe, pose no threat to humans, animals, plants, or earthworms, and thus offer a viable alternative to chemical pesticides [31]. EPNs go through complex processes to determine the suitability of a host [32]. Beyond host selection, successful infection by EPNs is influenced by factors such as the host’s immune response, population dynamics, and interactions with other organisms [33] Co-infection with other insect pathogens, including viruses, bacteria, other nematodes, or hymenopteran parasitoids, can enhance the success rate of these infections [34]. The interaction between nematodes and parasitoids favored the nematodes when they were introduced during the parasitoid’s young larval stage, enhancing the nematodes’ growth and reproduction, leading to the death of the parasitoid larvae. However, when nematodes were introduced during the parasitoid’s late larval stage, the competition somewhat favored the wasps, allowing about 50% of them to develop, emerge, and reproduce. This indicates that both nematodes and wasps can reproduce in the same host [35,36].

Insects encounter various stressors, both chemical (e.g., pesticides, heavy metals) and physical (e.g., radiation, temperature), leading to oxidative stress and cellular damage [37,38,39]. To combat reactive oxygen species (ROS) and prevent cellular damage, insects activate antioxidant enzymes (such as glutathione peroxidase (GPx), glutathione S-transferase (GST), and superoxide dismutase (SOD)) [40,41]. Malondialdehyde (MDA) is a common marker of oxidative stress, while GPx, GSTs, and SODs play critical roles in detoxification and antioxidative defense [42,43,44,45]. Cytochrome P450 enzymes, part of a heme-thiolate protein superfamily, are crucial for insect oxidative metabolism and detoxification [46,47]. They are involved in the metabolism of ecdysterone, juvenile hormones, and cuticular hydrocarbons, which are essential for insect development and growth [48].

Annexins, a large protein family, are involved in essential biological functions such as calcium metabolism, cell adhesion, growth, differentiation, subcellular transport, and membrane repair [49,50]. Flow cytometry using Annexin-V is a common method for detecting apoptosis at the cytological level [51]. Genotoxicity assays in insects, such as the comet assay, are valuable tools for studying DNA damage mechanisms and monitoring environmental contaminants [52]. The comet assay is a sensitive and reliable method for detecting DNA damage induced by physical agents or xenobiotics in insect tissues [53,54].

Building on our understanding of the biological control agents and the host response mechanisms, this study aims to comprehensively investigate the physiological, molecular, and morphological effects of the entomopathogenic nematode *Steinernema carpocapsae* (Weiser) (Rhabditida: Steinernematidae) (all strains) and the ectoparasite *B. hebetor* on *G. mellonella* larvae. Both individual and combined effects will be examined. This research offers a unique perspective by utilizing scanning electron microscopy to elucidate the morphological alterations induced by these bioagents. Additionally, we will explore the physiological and molecular changes occurring in the host larvae, providing a holistic understanding of these interactions.

## 2. Materials and Methods

### 2.1. Insect and Nematode Cultures

**Figure 1:**
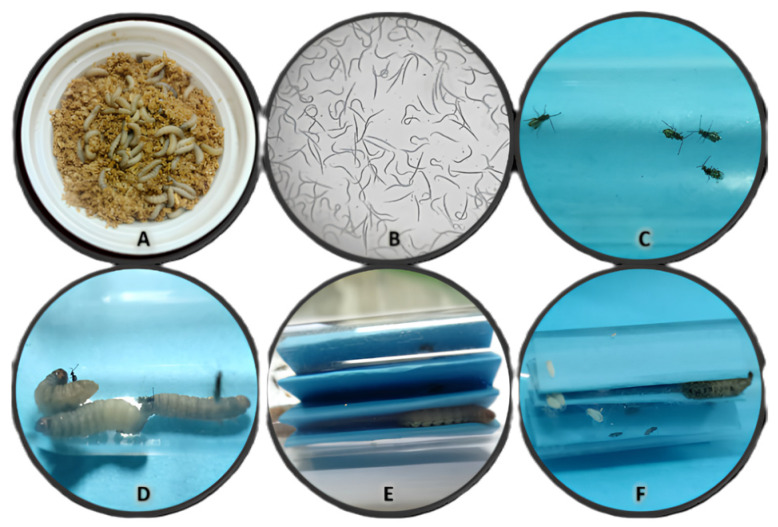
*Galleria mellonela* rearing (**A**), culture of parasites: *Steinernema carpocapsae* (**B**) and *Bracon hebetor* (**C**), and parasitism by *Bracon hebetor* (B.h.) on *Galleria mellonella* larvae (**D**–**F**).

Greater wax moth (*Galleria mellonella*): Larvae were obtained from infested beehives at the main farm of Alexandria University’s Faculty of Agriculture (31.2001° N latitude and 29.9187° E longitude). A laboratory colony was established and maintained at 25 °C with 50–70% relative humidity (RH) in complete darkness. Larvae were reared on a combined diet of honeycombs and an artificial mixture containing wheat flour (2.5 kg), wheat bran (2.5 kg), honey (750 g), medical dried yeast (500 g), low-fat milk powder (350 g), glycerin (800 g), methyl p-hydroxybenzoate (4 g), and freeze-dried beeswax powder (250 g), according to the methods described by Singh (1994) [55] (Figure 1A).

Entomopathogenic nematode (EPN)—*Steinernema carpocapsae* (all strains): Cultures were acquired from Dr. R. Gaugler’s laboratory at Rutgers University, New Jersey, USA. These cultures were maintained in the laboratory using *G. mellonella* larvae following the methods outlined by Woodring (1988) [56]. Specifically, last instar *G. mellonella* larvae were used for mass rearing the nematodes. Infective juveniles (IJs) of *S. carpocapsae* were harvested using White traps [57] at 25 ± 1 °C and stored at 10 °C until needed. Only IJs less than two weeks old were utilized for experiments (Figure 1B).

Parasitoid wasp—*Bracon hebetor*: The *B. hebetor* strain used originated from wild adults that emerged from parasitized larvae of *Plodia interpunctella* (Hubner) (Lepidoptera: Pyralidae). A laboratory colony was established and maintained on late-stage (25-day-old) *G. mellonella* larvae at 25 °C with 50–70% RH. Larvae, pupae, and adult wasps were initially collected from infested beehives at Alexandria University’s main farm. The host and parasitoid cultures were maintained in separate glass jars, with *B. hebetor* reared under a 12:12-h light/dark photoperiod (Figure 1C).

Experiment 1: Parasitism by *B. hebetor* (B.h.) on *G. mellonella* larvae.

Mating and parasitism: Newly emerged (two-day-old) male and female *B. hebetor* wasps (10 per group) were paired in glass vials (2 cm × 10 cm) for mating over 24 h. Last-instar *G. mellonella* larvae (20 larvae per group) were placed in separate Petri dishes (8.5 cm × 1.3 cm). Each dish was replicated three times and furnished with small pieces of corrugated cardboard to provide shelter for the larvae. After allowing the larvae to settle for 2–3 h, mated female wasps (2 days old, 5 per dish) were introduced and allowed to parasitize the hosts. Four days later, the parasitoid larvae were collected for subsequent analyses (Figure 1D–F).

Experiment 2: Parasitism by *S. carpocapsae* (S.c.) nematodes on *G. mellonella* larvae.

Nematode exposure: Twenty late-instar *G. mellonella* larvae were introduced to individual plastic Petri dishes (9 cm × 1.5 cm) previously inoculated with *S. carpocapsae* nematodes (100 ij/larva). The dishes were then incubated for 24 h in a climate-controlled chamber (ICB-CC-3 Series) maintained at 25 °C, 50–70% relative humidity (RH), and a 16:8 h light/dark cycle. Following incubation, the larvae were transferred to cups and collected after 48 h for various assessments.

Experiment 3: Combined parasitism by *B. hebetor* and *S. carpocapsae* (B.h. + S.c.) on *G. mellonella* larvae.

Sequential parasitism: *G. mellonella* larvae that had been parasitized by *B. hebetor* for two days were then transferred to dishes inoculated with *S. carpocapsae* nematodes. These dishes were incubated for 24 h under the same conditions as Experiment 2 (25 °C, 50–70% RH, 16:8-h light/dark cycle). Larvae exposed to combined parasitism were collected after three days for assessment. A control group consisted of *G. mellonella* larvae placed in Petri dishes and inoculated with 1 mL of distilled water. Neither nematodes nor *B. hebetor* were introduced to the control group.

### 2.2. Sample Processing and Analysis

Twenty larvae from each control and parasitism experiment were designated for various analyses. Half of the larvae were stored at −80 °C in vials for subsequent biochemical and molecular analyses. The remaining larvae were immediately fixed in 4% formaldehyde and 1% glutaraldehyde (4F1G) in 0.1 M phosphate buffer (pH 7.2) at 4 °C for three hours. These fixed larvae were then post-fixed with 2% osmium tetroxide (OsO_4_) in the same buffer for two hours to prepare them for SEM.

For enzymatic assays, both treated and control larvae were homogenized in Tris-HCl buffer (pH 7.4) at a tissue-to-buffer ratio of 1:5 for 2 min. The homogenate was then centrifuged at 15,000× *g* for 60 min at 4 °C. The resulting supernatant was carefully collected and stored cold until further analysis.

### 2.3. Biochemical Analyses

MDA content was determined following the method described in [58]. The reaction mixture, containing supernatant, 8% sodium dodecyl sulfate (SDS), 20% acetic acid, 0.8% thiobarbituric acid (TBA), and ultrapure water, was prepared in a 1:2:15:15:7 (*v*/*v*/*v*/*v*/*v*) ratio. This mixture was then incubated at 95 °C for one hour. After cooling, the sample was mixed with ultrapure water and butanol-pyridine (15:1 *v*/*v*), shaken for 10 min, and centrifuged at 15,000× *g* for 10 min. The absorbance of the top butanol-pyridine layer was measured at 532 nm. The MDA content was expressed as nmol/mg of tissue.

Antioxidant enzyme activities: 

GPx activity was determined using the method described in [59], with cumene hydroperoxide (cumOOH) as the substrate. The reaction mixture contained the prepared supernatant, 1.25 mM EDTA, 1 mM sodium azide (NaN_3_, to inhibit catalase activity), 1 unit (IU) of glutathione reductase, 0.05 M phosphate buffer (pH 7.0), and 0.1 mM NADPH. The reaction was initiated by adding 0.2 mM cumOOH, and the absorbance was measured at 340 nm for two minutes. GPx activity was expressed as mU/mg protein.

GST activity was assessed following the methods outlined in [60,61], using 1-chloro-2,4-dinitrobenzene (CDNB) as the substrate. The reaction mixture contained the prepared supernatant, 33 mM Tris-HCl buffer (pH 9.0), and 7.5 mM GSH. The reaction was initiated by adding 15 mM CDNB, and the formation of the glutathione–CDNB complex was monitored spectrophotometrically at 340 nm. GST activity was expressed as mU/mg protein.

SOD activity was measured according to the method described in [62], which involves monitoring the auto-oxidation rate of adrenaline. The SOD assay utilized a reaction mixture containing the prepared supernatant and a 200 mM sodium carbonate buffer at pH 10.0. SOD activity was quantified in mU/mg based on the reaction mixture’s absorbance.

Cytochrome P450 activity was determined using insect homogenates according to the method described in [63]. The activity was expressed as milliunits of cytochrome P450 per milligram of protein (mU/mg protein).

### 2.4. DNA Damage Assessment

Comet assay: DNA damage was assessed using the comet assay in an alkaline environment, following the protocol established by Singh et al. (1988) [64]. Testicular cells were isolated by gently macerating the tissue in cold Hank’s balanced salt solution containing 10% dimethyl sulfoxide (DMSO) and 20 mM ethylenediaminetetraacetic acid (EDTA). The cell suspension was mixed with low-melting-point agarose and applied to microscope slides. After solidification, the slides underwent lysis, rinsing, and electrophoresis steps to separate damaged DNA fragments. The slides were then stained with a fluorescent dye, and comets (indicators of DNA damage) were analyzed under a microscope. One hundred comets per slide were scored for analysis.

### 2.5. Cell Viability Evaluation

Annexin V-FITC assay: Cell viability was evaluated using a commercially available Annexin V-FITC test kit (Sigma-Aldrich, Darmstadt, Germany) according to the recommendations of the manufacturer. The tissue was homogenized in a cold PBS buffer (pH 7.4), and the cell suspension was washed and resuspended in a binding buffer. The suspension was then incubated with Annexin V-FITC conjugate, a fluorescent marker for apoptotic cells, and propidium iodide, a marker for necrotic cells. Fluorescence intensity was measured using flow cytometry to assess cell viability and the extent of cell death (apoptosis and necrosis). Data were analyzed using Cell Quest Pro software version 5.2.1 (BD Biosciences, San Jose, CA, USA).

### 2.6. Morphological Analysis

Morphological differences between *G. mellonella* larvae from different treatment groups were examined using a JEOL JSM-5300 SEM (JEOL Ltd., Tokyo, Japan) at the Electron Microscope Unit (EMU) of Alexandria University’s Faculty of Science, Egypt. Last-instar larvae were prepared for SEM analysis by following a fixation, post-fixation, and dehydration protocol involving an ethanol series.

### 2.7. Statistical Analysis

All data were statistically analyzed to ensure validity and identify significant differences between groups. Homogeneity of variance, a crucial assumption for analysis of variance (ANOVA), was assessed using Levene’s test. Normality of the data distribution was assessed using the Kolmogorov–Smirnov and Lilliefors tests. Based on these tests, a one-way ANOVA followed by Tukey’s post hoc test (*p* < 0.05) was chosen for groups with equal sample sizes. Mean values and standard deviations (SDs) were presented in the figures, with identical letters indicating groups that were not statistically different from each other based on the Tukey’s test. Additionally, principal component analysis (PCA) was employed to explore potential correlations and patterns among the various biochemical parameters measured in the study. Statistical analysis was performed using Statistica 13.3 software.

## 3. Results

### 3.1. Stress Biomarkers

**Figure 2:**
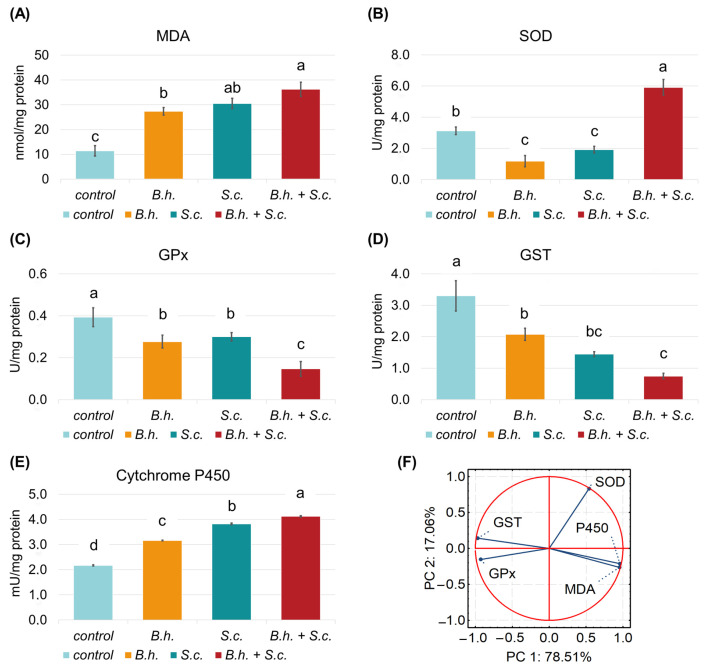
Mean ± SD of (**A**) Malondialdehyde (MDA) content and activity of (**B**) Superoxide dismutase (SOD), (**C**) Glutathione peroxidase (GPx), (**D**) Glutathione S-transferase (GST), and (**E**) Cytochrome P450, as well as (**F**) Principal Component Analysis (PCA) on all variables measured in larvae of *Galleria mellonella* from the control group or those infected separately with the parasites *Bracon hebetor* (B.h.) or nematode *Steinernema carpocapsae* (S.c.) or combined parasitism (B.h. + S.c.). Groups with the same letters indicate no significant difference between them (Tukey’s post hoc, *p* < 0.05).

Significant differences were observed in stress biomarkers among the different treatment groups. The final product of polyunsaturated fatty acid peroxidation, MDA, was three times higher in larvae from the combination group (B.h. + S.c.) (36.21 nmol/mg protein) compared to the control group (11.4 nmol/mg protein) (Figure 2A). Conversely, SOD activity, an enzyme crucial for converting superoxide into hydrogen peroxide, was found to be lower in the B.h. and S.c. groups (1.18 and 1.92 U/mg protein, respectively) but highest in the combination group (5.91 U/mg protein) (Figure 2B). The activities of GPx and GST were higher in the control larvae (0.39 and 3.3 U/mg protein, respectively) compared to the infected groups (Figure 2C,D).

### 3.2. Cytochrome P450 and Principal Component Analysis (PCA)

Cytochrome P450 enzymes, linked to detoxification in insects, showed notable activity in response to *G. mellonella* parasitism (Figure 2E). PCA analysis revealed that the first two principal components (PC1 and PC2) explained 95.57% of the data variability. PC1 explained 78.51%, while PC2 accounted for 17.06%. PC1 displayed a negative correlation with GST and GPx activity, while SOD and, cytochrome P450 activity and MDA content positively correlated with PC1 (Figure 2F).

**Figure 3:**
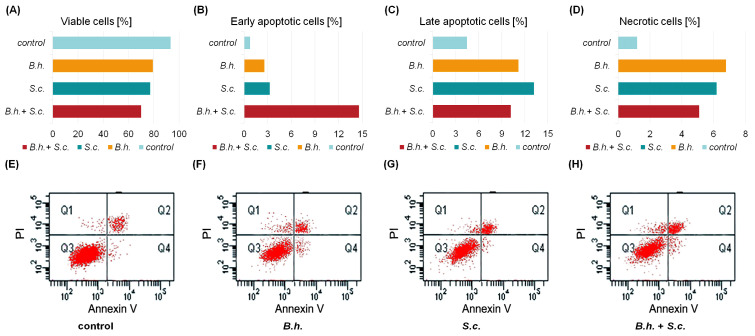
The percentages of (**A**) viable, (**B**) early apoptotic, (**C**) late apoptotic, and (**D**) necrotic cells, determined using the Annexin V-FITC test kit in larvae of *Galleria mellonella* from the control group or those infected separately with the parasites *Bracon hebetor* (B.h.) or Nematode *Steinernema carpocapsae* (S.c.), or combined parasitism (B.h. + S.c.). (**E**–**H**) examples of cytometric plots for control and experimental groups. The diagram of flow cytometric analysis was divided into four quadrants: necrotic cells (Q1: upper left quadrant), late apoptotic cells (Q2: upper right quadrant), living cells (Q3: lower left quadrant), and early apoptotic cells (Q4: lower right quadrant).

Cell Death Analysis: Following staining with Annexin V-FITC and propidium iodide, flow cytometric analysis of *G. mellonella* cells from the B.h., S.c., and B.h. + S.c. groups demonstrated a significant increase in the proportion of apoptotic cells, particularly those exhibiting early apoptotic characteristics (Figure 3A–D). The proportion of cells undergoing late apoptosis and necrosis was also higher in parasitized insects compared to the control group.

Parasites reduced the viability of cells, as shown by the Annexin V-FITC assay (Figure 3E–H). Compared to the control group, the percentages of viable cells in the B.h., S.c., and B.h. + S.c. groups were 79.4%, 77.3%, and 70.1%, respectively. Differences were also observed in the percentages of necrotic and apoptotic cells between the groups. Insect groups with parasites had more necrotic and apoptotic cells compared to the control group. Necrosis rose from 1.2% in the control group to 6.8% in the B.h. group. Early apoptosis jumped from 0.8% in the control to 14.6% in the B.h. + S.c. group, and late apoptosis increased from 4.5% in the control to 13.2% in the S.c. group.

**Figure 4:**
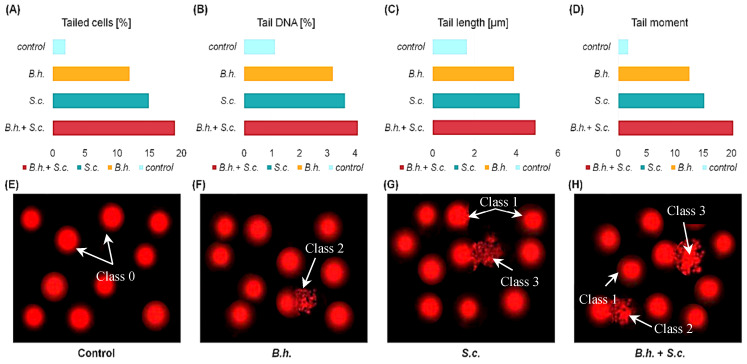
The percentages of (**A**) tailed cells, (**B**) amount of DNA in the comet tail, (**C**) tail length, and (**D**) tail moment, measured by the Comet assay in larvae of *Galleria mellonella* from the control group or those infected separately with the parasites *Bracon hebetor* (B.h.) or Nematode *Steinernema carpocapsae* (S.c.) or combined parasitism (B.h. + S.c.). (**E**–**H**) examples of DNA-damaged nuclei for control and experimental groups.

DNA Damage Analysis: DNA damage was increased in the B.h.-, S.c.-, and B.h. + S.c.-treated groups compared to the control group, as evidenced by the strand breaks appearing as comet tails in the comet assay (Figure 4E–H). Additionally, the DNA-damaged cells classified in classes 1, 2, and 3 were more prevalent in the treated groups compared to the control (class 0).

Tail length (TL) fluorescence increased in classes 2 and 3 of the treated groups. Classes 1, 2, and 3 around the head indicated the frequency of cell death. In the control comet cells (class “0”), DNA migration from the nucleoid core was negligible (Figure 4A–D). Analysis of DNA damage revealed clear differences between insects from different treatments (Figure 4). In control insects, the amount of DNA in the comet tail (TDNA) did not exceed 1.12%, and the average tail length (TL) was just under 2 µm, indicating minimal damage. In contrast, insects from the treated groups exhibited significant DNA damage, reflected in the amount of fragmented DNA in the comet tail (mean TDNA ranged from 3.22% to 4.12%) and tail length (mean TL ranged from 3.91 µm to 4.94 µm).

**Figure 5:**
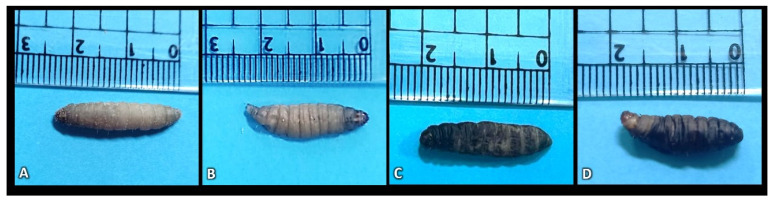
Observed color changes in larvae of *Galleria mellonella*: (**A**) control larva with normal appearance characterized by light creamy color, (**B**) B.h. group (creamy), (**C**) S.c. group (dark crimson), and (**D**) B.h. + S.c. group (black).

Morphological Analysis of *G. mellonella* Larvae: The final larval instar of the Greater Wax Moth (*G. mellonella*) was infected with the entomopathogenic nematode *S. carpocapsae* and the ectoparasite *B. hebetor*, both individually and in combination. The morphology of these treated larvae was then compared to a control group. Figure 5 illustrates the observed larval aberrations following infection. The control larva (Figure 5A) displayed a normal appearance with a light creamy color, similar to the B.h. group larvae (Figure 5B). In contrast, larvae in the S.c. group exhibited shrinkage and a dark crimson coloration (Figure 5C), while those in the B.h. + S.c. group shrank and turned black (Figure 5D).

**Figure 6:**
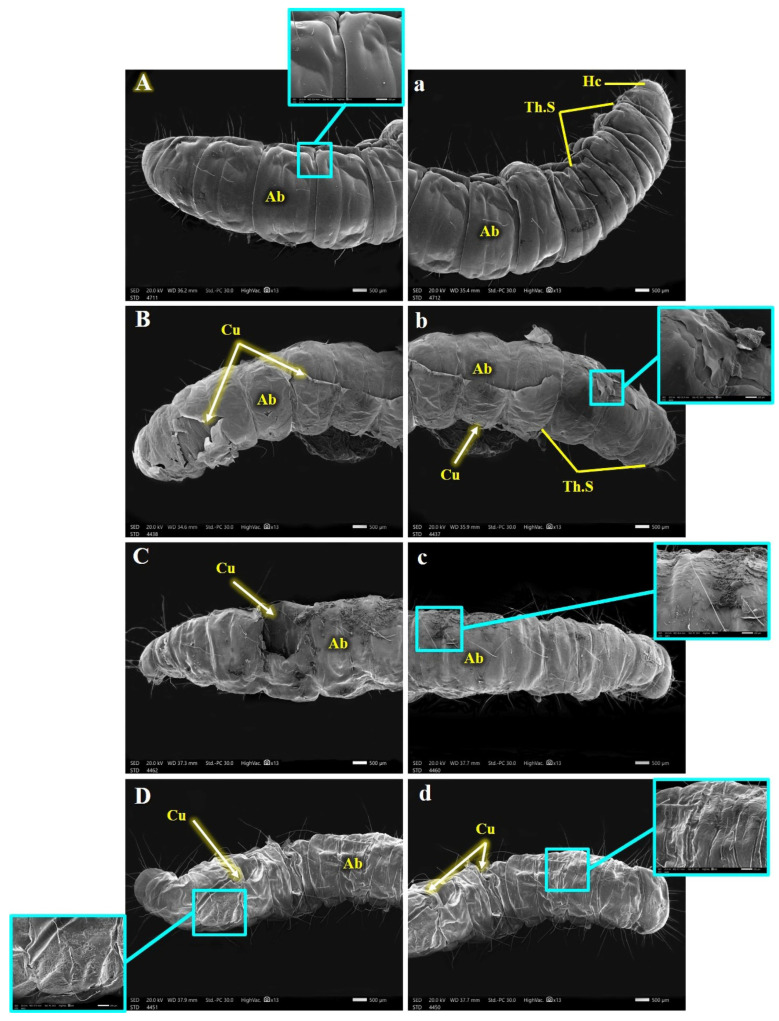
SEM photography for dorsal anterior view (**a**–**d**) and the dorsal posterior end (**A**–**D**) of control and parasitic *Galleria mellonella* last instar larvae, showing: Hc, Head capsule; Th.S, Thorax segments; Ab, Abdomen; Cu, cuticle.

**Figure 7:**
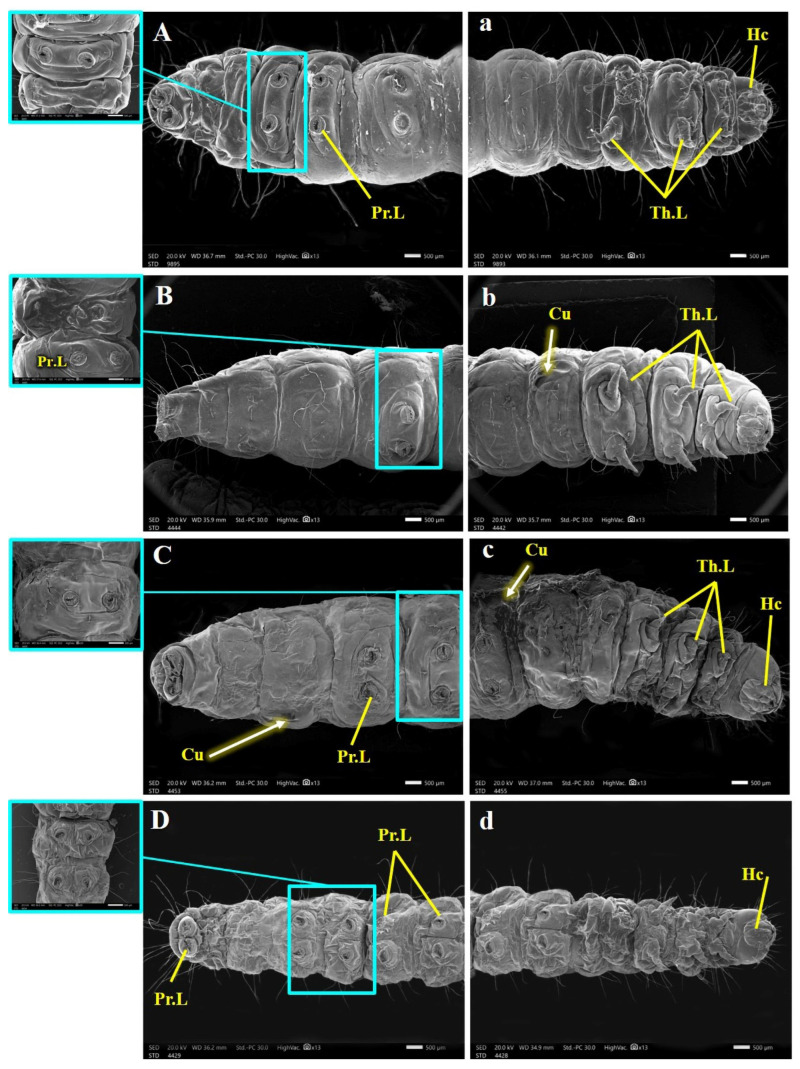
SEM photography for anterior ventral view (**a**–**d**) and posterior ventral view (**A**–**D**) of control and parasitic *Galleria mellonella* last instar larvae, showing: Hc, head capsule; Th.L, thoracic lags; Pr.L, abdominal prolegs; Cu, cuticle.

**Figure 8:**
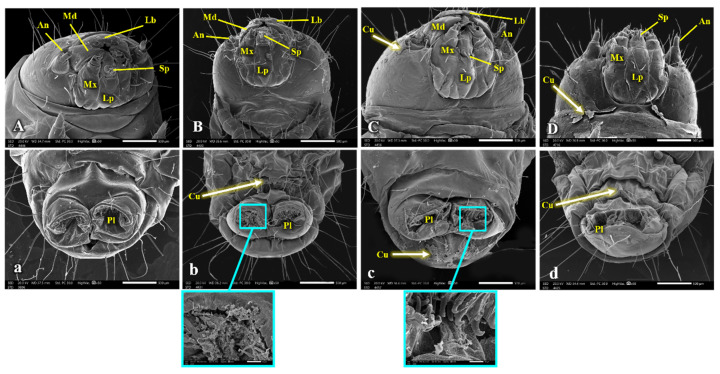
SEM photography for head capsule (**A**–**D**) and posterior end (**a**–**d**) of the control and parasitic *Galleria mellonella* last larval instar, showing: An, antenna; Lr, labrum; Lp, labium; Md, mandible; Mx, maxilla; Sp, spinneret; Cu, cuticle; Pl, abdominal prolegs.

Uninfected *G. mellonella* Larval Morphology (Figure 6, Figure 7 and Figure 8): Figure 6, Figure 7 and Figure 8 depict the morphology of the uninfected last larval instar of *G. mellonella*. These larvae possess a hardened head capsule (sclerotized). The thorax is composed of three segments, each bearing a pair of segmented legs. Additionally, a pair of spiracles (breathing pores) can be found on the lateral sides (pleura) of the prothorax and metathorax segments. The abdomen consists of ten segments, each with a hardened upper plate (tergum) and a lower plate (sternum) connected by a membranous pleural region. The first eight abdominal segments each have a pair of spiracles, while segments 3, 4, 5, 6, and 10 have five pairs of prolegs (fleshy appendages) ending in hooks (crochets) used for locomotion.

### 3.3. SEM Analysis

SEM revealed several abnormalities and malformations in the cuticle and associated sensilla of *G. mellonella* larvae from the treated groups (B.h., S.c., and B.h. + S.c.) compared to the control group. Obvious distortions and damage to the cuticle were observed in various body segments (Figure 6, Figure 7 and Figure 8).

Figure 6 presents SEM photomicrographs showcasing the dorsal morphology of the larvae. Panels a-d depict the dorsal anterior view, while panels A-D represent the dorsal posterior end. Unlike the smooth cuticles in the control group, the B.h. and S.c. groups had ridges and tears on their tergum cuticle (Figure 6B,b,C,c). Notably, larvae in the B.h. + S.c. group displayed a rough and wrinkled cuticle (rugose) (Figure 6D,d).

The ventral side of the larvae also exhibited numerous abnormalities as revealed by SEM analysis (Figure 7). These abnormalities included a ripped sternum cuticle, a ridged cuticular surface, disorganized hair-like sensory structures (trichoid sensilla), and indistinct segmentation.

Figure 8 focuses on the head capsule and caudal segment of the larvae. Observations of the caudal segment revealed sunken prolegs and missing crochets (Figure 8d), along with a ruptured sternum cuticle at the abdominal end (Figure 8c). Additionally, the SEM images show that the cuticle of the head capsule shed separately from the rest of the body (Figure 8C,D).

## 4. Discussion

The greater wax moth, *G. mellonella*, is a major global pest of beehives. Its larvae are also widely used as a model organism to study insect infections and evaluate the effectiveness of biocontrol agents. This study provides valuable insights into the molecular mechanisms underlying *G. mellonella*’s responses to parasitic infections by the idiobiont ectoparasitoid *B. hebetor* and the entomopathogenic nematode *S. carpocapsae*. Understanding the ecophysiological effects of *B. hebetor* on pest insects’ metabolism, immunity, and genome is crucial for developing environmentally friendly bioinsecticides. Such knowledge can contribute to sustainable integrated biological control strategies for managing *G. mellonella* populations [65].

Insects rely on antioxidant metabolism to maintain physiological homeostasis and respond to various stress stimuli, including parasitoid attack. Parasites generate reactive oxygen species (ROS) that lead to oxidative stress and, tissue damage, and exacerbate infection pathology. Nematode attacks can also cause significant metabolic stress in host insects by inducing enzymatic changes [66]. In response to these stressors, *G. mellonella* typically increases the activity of antioxidant enzymes such as SOD, GST, and GPx. Studies have shown that parasitoids can affect these enzymes in their host insects, influencing their immune and defense systems [67,68].

Oxidative stress in biological systems arises from superoxide anion radicals, hydroxyl radicals, and hydrogen peroxide produced during normal cellular activities. These ROS are toxic to cellular processes, but their role in insect immune defense is becoming increasingly recognized. Our investigation found a significant rise in SOD levels six hours after infection, which aligns with previous findings [69]. SOD plays a critical role in mitigating oxidative stress induced by various stressors and is involved in the innate immune response of many invertebrates [70].

Our results demonstrate that parasitization by *B. hebetor* and *S. carpocapsae*, both individually and in combination (B.h. and S.c. groups), led to a downregulation of SOD, GPx, and GST activity compared to control larvae. These findings are consistent with previous studies suggesting changes in antioxidant gene levels following parasitism [71,72]. For example, Shafeeq et al. (2017) [73] reported that envenomation by *B. hebetor* downregulated SOD activity in the host *Plodia interpunctella*. Similarly, parasitization by *P. turionellae* significantly decreased SOD activity in host hemolymph [74].

GPx detoxifies lipid peroxides and protects cell membranes from oxidative damage. Our results regarding GPx activity differ from those of Duman Erbas & Altuntas (2021) [75], who observed no significant changes in GPx activity in *G. mellonella* larvae treated with juglone. GST plays another crucial role in insect detoxification. While our study showed increased GST activity in control larvae compared to infected groups, Çim & Altuntaş (2021) [74] reported a decrease in GST activity in parasitized host hemolymph. These discrepancies highlight the potential influence of different parasitoid species, venom composition, and experimental conditions on host insect antioxidant enzyme regulation. Interestingly, some studies suggest that idiobiont endoparasitoid venom may reduce oxidative stress in the host-parasite system, potentially explaining the observed downregulation of antioxidant enzymes in our study. MDA concentration, a byproduct of lipid peroxidation and a marker of oxidative stress, was significantly higher in the combination (B.h. + S.c.) group compared to control larvae. This finding suggests increased oxidative damage in response to the combined parasitism. It contrasts with the decreased MDA levels reported by Çim & Altuntaş (2021) [74] following parasitization by *P. turionellae*, highlighting potential differences in host responses depending on the specific parasite species. Additionally, studies have shown that force-feeding okadaic acid to *G. mellonella* larvae increases MDA levels, leading to tissue damage and oxidative stress [67].

Our study also revealed activation of cytochrome P450 enzymes in response to *G. mellonella* parasitism. This aligns with the findings of Rewitz et al. (2006a, 2006b, 2006c) [76,77,78], who demonstrated the role of P450s in insect detoxification. However, the specific activities of these enzymes in parasitoid wasps remain understudied. Notably, research indicates that conserved P450s often play developmental roles, such as those involved in 20E biosynthesis in *Drosophila melanogaster* and *Manduca sexta*.

Annexins are multifunctional proteins with roles in both cell biology and pathology. They can be expressed in parasite structures and secretions, as well as in host cells infected by parasites [79]. Our results, demonstrating increased apoptotic cells in parasitized groups, support findings that suggest annexin targeting can influence host defense during infections [80]. Targeting or inhibiting these proteins may have various consequences during illnesses and infections, including parasitic diseases. Potential benefits include reducing infection-induced acute inflammation or enhancing host defense mechanisms [80,81].

DNA damage resulting from parasitism can be evaluated using the comet assay, which analyzes tail migration to assess the extent of DNA strand fragmentation. Studies on the DNA-damaging effects of various parasites on insects using this technique are limited. Our study demonstrates that parasitism by *B. hebetor*, *S. carpocapsae*, or their combination induces different levels of DNA damage in *G. mellonella* larvae. These findings are consistent with previous work by Çim & Altuntaş (2021) [74] who investigated the genotoxic effects of *P. turionellae* venom on *G. mellonella*. The study showed that wasp venom damaged the DNA of host pupae more severely at all tested concentrations. This damage increased over time. The results suggest that wasp venom creates oxidative stress in the host, harming its enzymes and DNA. These changes might create a suitable environment for the wasp larvae to develop successfully.

Chitin, a polymer of N-acetyl-D- glucosamine, is a major component of the insect cuticle and is primarily produced by nematodes and constitutes a large portion of the insect cuticle. Breaches in these cuticular barriers trigger the activation of *G. mellonella*’s defensive mechanisms. Nematode infective juveniles (IJs) typically penetrate the host’s body through natural openings or areas with a thinner cuticle [82]. Our SEM results revealed that parasitism by *B. hebetor*, *S. carpocapsae*, or their combination (B.h. + S.c.) caused qualitative differences in the external morphology between control and affected *G. mellonella* larvae. These results support previous research, which indicates that parasitization by *B. hebetor* and entomopathogenic nematodes can cause morphological abnormalities in the host cuticle. In response to nematode infection, insect pests have evolved various morphological and immunological defense strategies [83]. *G. mellonella* relies on a chitin-based integument for protection. This integument also shields vital ectodermal organs like the trachea, foregut, and hindgut from infections [84].

## 5. Conclusions

**Figure 9:**
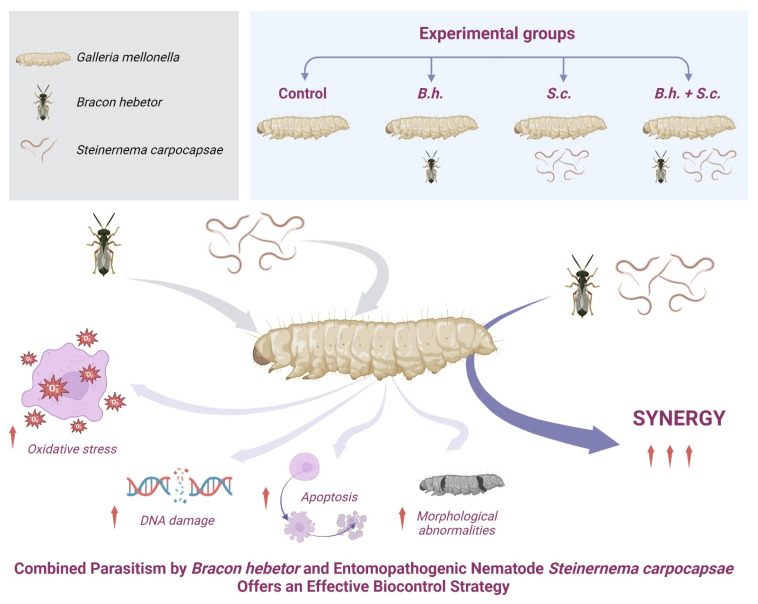
Summary of the effects of *Bracon hebetor* and *Steinernema carpocapsae* on *Galleria mellonella*.

Entomopathogenic nematodes and parasitoid wasps play a vital role in biological pest control strategies. This study has demonstrated significant physiological, molecular, and morphological impacts on *G. mellonella* larvae following parasitism by *B. hebetor*, *S. carpocapsae*, or both (B.h. + S.c.). These findings not only validate previous research on the individual effects of these biocontrol agents but also highlight the potential of a novel and effective strategy: the combined parasitism of *B. hebetor* and entomopathogenic nematodes for controlling *G. mellonella* populations (integrated pest management approach) (Figure 9). This combined approach presents a promising path for developing sustainable and eco-friendly pest management practices.

## Data Availability

Data is contained within the article.
